# Near-Complete Genome Sequence of the Cellulolytic Bacterium *Bacteroides* (*Pseudobacteroides*) *cellulosolvens* ATCC 35603

**DOI:** 10.1128/genomeA.01022-15

**Published:** 2015-09-24

**Authors:** Bareket Dassa, Sagar Utturkar, Richard A. Hurt, Dawn M. Klingeman, Martin Keller, Jian Xu, Y. Harish Kumar Reddy, Ilya Borovok, Inna Rozman Grinberg, Raphael Lamed, Olga Zhivin, Edward A. Bayer, Steven D. Brown

**Affiliations:** a Department of Biological Chemistry, Weizmann Institute of Science, Rehovot, Israel; b Graduate School of Genome Science and Technology, University of Tennessee, Knoxville, Tennessee, USA; c BioEnergy Science Center, Oak Ridge, Tennessee, USA; d Biosciences Division, Oak Ridge National Laboratory, Oak Ridge, Tennessee, USA; e Single-Cell Center, CAS Key Laboratory of Biofuels and Shandong Key Laboratory of Energy Genetics, Qingdao Institute of Bioenergy and Bioprocess Technology, Chinese Academy of Sciences, Qingdao, Shandong, China; f Department of Molecular Microbiology and Biotechnology, Tel Aviv University, Ramat Aviv, Israel

## Abstract

We report the single-contig genome sequence of the anaerobic, mesophilic, cellulolytic bacterium, *Bacteroides cellulosolvens*. The bacterium produces a particularly elaborate cellulosome system, wherein the types of cohesin-dockerin interactions are opposite of other known cellulosome systems: cell-surface attachment is thus mediated via type-I interactions, whereas enzymes are integrated via type-II interactions.

## GENOME ANNOUNCEMENT

The cellulosome is one of the most efficient systems known to biodegrade plant cell-wall polysaccharides and cellulosic wastes. This multi-enzyme extracellular complex incorporates multiple hydrolytic enzymes onto the bacterial cell-surface through dockerin modules that tightly bind to scaffoldin proteins via complementary cohesin modules (1–3). Additional carbohydrate-binding modules (CBM) attach the entire enzymatic complex to the cellulosic substrate (4). The biodegrading activity of cellulosomes has been studied extensively in related cellulolytic bacteria, such as *Clostridium (Ruminiclostridium) thermocellum, Acetivibrio cellulolyticus, Clostridium (Ruminiclostridium) clariflavum, Clostridium (Ruminiclostridium) cellulolyticum*, *Clostridium cellulovorans, Clostridium (Ruminiclostridium) papyrosolvens*, and *Ruminococcus flavefaciens* (5).

*Bacteroides cellulosolvens* ATCC 35603 (DSM 2933) is a cellulolytic bacterium, originally isolated from sewage sludge (6, 7) in co-culture with *Clostridium saccharolyticum*. Initially classified as a Gram-negative bacterium, analysis of the 16S RNA indicated that *B. cellulosolvens*, like *A. cellulolyticus*, is a member of the phylogenetically diverse clostridial assemblage (8, 9). Recently, *B. cellulosolvens* was renamed *Pseudobacteroides cellulosolvens* (10).

*B. cellulosolvens* was selected for its ability to grow under mesophilic, anaerobic conditions, and the bacterium was able to bind and degrade crystalline cellulose to cellobiose and glucose (11–13). Its cellulose-degrading activity was shown to be cell-associated (14), and elaborate cellulolytic cell-surface structures were subsequently demonstrated (15, 16). Cellulosome-like complexes were further identified in the bacterium (17), supported by the recognition of the major scaffoldin protein (CipBc, later renamed ScaA) (18, 19), which includes eleven type-II cohesin domains, a family-3a CBM, and a C-terminal dockerin domain. Its scaffoldin was shown to interact with a family-48 glycoside hydrolase (18), and the crystal structure of its type-II cohesin was determined (20).

The genome is reported as a large contig of 6,878,816 bp, translated into 5,897 predicted proteins. Sequencing was performed using PacBio RS-II technology and data from four SMRT cells was assembled using SMRTanalysis v2.2 (HGAP3 protocol). The initial assembly generated three contigs at ~65× raw read coverage, which were joined using Geneious R8 (21) and then validated by PCR and Sanger sequencing (22). Illumina reads (at ~200× coverage) also confirmed contiguity. The ends of the single contig were unable to be joined experimentally or *in silico*, possibly as a result of a misassembly or active mobile genetic element. Active transposase systems have been shown to interfere with closure previously (23). Therefore, the genome is reported as near-complete assembly. Gene prediction and annotation were performed as described previously (24, 25).

Intriguingly, the types of cohesin-dockerin interaction in *B. cellulosolvens* are reversed from those of all other known cellulosome systems, whereby cell-surface attachment of noncatalytic scaffoldins in *B. cellulosolvens* is mediated via type-I interactions, whereas the enzymes are integrated via type II-interactions (19, 26, 27). The genome codes for 75 cohesin modules (mostly type-II cohesins), packaged in more than two-dozen scaffoldins, and over 200 dockerin-containing proteins, including glycoside hydrolases, carbohydrate esterases, and polysaccharide lyases. Thus, *B. cellulosolvens* scaffoldins represent the largest noncatalytic cellulosomal subunits known to date, indicating the presence of a particularly elaborate cellulosome system.

### Nucleotide sequence accession numbers.

This whole-genome shotgun project has been deposited at DDBJ/EMBL/GenBank under the accession number LGTC00000000. The version described in this paper is version LGTC01000000.

## References

[1] Bayer EA, Morag E, Lamed R 1994 The cellulosome—A treasure-trove for biotechnology. Trends Biotechnol 12:379–386. doi:10.1016/0167-7799(94)90039-6. 7765191

[2] Bayer EA, Belaich J, Shoham Y, Lamed R 2004 The cellulosomes: Multienzyme machines for degradation of plant cell wall polysaccharides. Annu Rev Microbiol 58:521–554. doi:10.1146/annurev.micro.57.030502.091022. 15487947

[3] Fontes CM, Gilbert HJ 2010 Cellulosomes: highly efficient nanomachines designed to deconstruct plant cell wall complex carbohydrates. Annu Rev Biochem 79:655–681. doi:10.1146/annurev-biochem-091208-085603. 20373916

[4] Boraston AB, Bolam DN, Gilbert HJ, Davies GJ 2004 Carbohydrate-binding modules: fine-tuning polysaccharide recognition. Biochem J 382:769–781. doi:10.1042/BJ20040892. 15214846 PMC1133952

[5] Bayer EA, Lamed R, White BA, Flint HJ 2008 From cellulosomes to cellulosomics. Chem Rec 8:364–377. doi:10.1002/tcr.20160. 19107866

[6] Murray WD, Sowden LC, Colvin JR 1984 *Bacteroides cellulosolvens* sp. nov., a cellulolytic species from sewage sludge. Int J Syst Bacteriol 34:185–187. doi:10.1099/00207713-34-2-185.

[7] Murray WD, Sowden LC, Colvin JR 1986 Symbiotic relationship of *Bacteroides cellulosolvens* and *Clostridium saccharolyticum* in cellulose fermentation. Appl Environ Microbiol 51:710–715. 16347034 10.1128/aem.51.4.710-714.1986 PMC238952

[8] Lin C, Urbance JW, Stahl DA 1994 *Acetivibrio cellulolyticus* and *Bacteroides cellulosolvens* are members of the greater clostridial assemblage. FEMS Microbiol Lett 124:151–155. doi:10.1111/j.1574-6968.1994.tb07277.x. 7813884

[9] Yutin N, Galperin MY 2013 A genomic update on clostridial phylogeny: gram-negative spore formers and other misplaced clostridia. Environ Microbiol 15:2631–2634. doi:10.1111/1462-2920.12173. 23834245 PMC4056668

[10] Horino H, Fujita T, Tonouchi A 2014 Description of *Anaerobacterium chartisolvens* gen. nov., sp. nov., an obligately anaerobic bacterium from *Clostridium* rRNA cluster III isolated from soil of a Japanese rice field, and reclassification of *Bacteroides cellulosolvens* Murray et al. 1984 as *Pseudobacteroides cellulosolvens* gen. nov., comb. nov. Int J Syst Bacteriol 64:1296–1303. doi:10.1099/ijs.0.059378-0. 24425743

[11] Giuliano C, Khan AW 1984 Cellulase and sugar formation by *Bacteroides cellulosolvens,* a newly isolated cellulolytic anaerobe. Appl Environ Microbiol 48:446–448. 16346612 10.1128/aem.48.2.446-448.1984 PMC241537

[12] Giuliano C, Khan AW 1985 Conversion of cellulose to sugars by resting cells of a mesophilic anaerobe, *Bacteroides cellulosolvens*. Biotechnol Bioeng 27:980–983. doi:10.1002/bit.260270708. 18553767

[13] Murray WD 1985 Increased cellulose hydrolysis by *Bacteroides cellulosolvens* in a simplified synthetic medium. J Biotechnol 3:131–140. doi:10.1016/0168-1656(85)90014-8.

[14] Murray WD, Sowden LC, Colvin JR 1986 Localization of the cellulase activity of *Bacteroides cellulosolvens*. Lett Appl Microbiol 3:69–72. doi:10.1111/j.1472-765X.1986.tb01550.x.

[15] Lamed R, Naimark J, Morgenstern E, Bayer EA 1987 Specialized cell surface structures in cellulolytic bacteria. J Bacteriol 169:3792–3800. 3301817 10.1128/jb.169.8.3792-3800.1987 PMC212468

[16] Morag E, Halevy I, Bayer EA, Lamed R 1991 Isolation and properties of a major cellobiohydrolase from the cellulosome of *Clostridium thermocellum*. J Bacteriol 173:4155–4162. 2061292 10.1128/jb.173.13.4155-4162.1991 PMC208065

[17] Lamed R, Morag (Morgenstern) E, Mor-Yosef O, Bayer EA 1991 Cellulosome-like entities in *Bacteroides cellulosolvens*. Curr Microbiol 22:27–33. doi:10.1007/BF02106209.

[18] Ding S-Y, Bayer EA, Steiner D, Shoham Y, Lamed R 2000 A scaffoldin of the *Bacteroides cellulosolvens* cellulosome that contains 11 type II cohesins. J Bacteriol 182:4915–4925. doi:10.1128/JB.182.17.4915-4925.2000. 10940036 PMC111372

[19] Xu Q, Bayer EA, Goldman M, Kenig R, Shoham Y, Lamed R 2004 Architecture of the *Bacteroides cellulosolvens* cellulosome: description of a cell-surface-anchoring scaffoldin and a family-48 cellulase. J Bacteriol 186:968–977. doi:10.1128/JB.186.4.968-977.2004. 14761991 PMC344227

[20] Noach I, Frolow F, Jakoby H, Rosenheck S, Shimon LJW, Lamed R, Bayer EA 2005 Crystal structure of a type-II cohesin module from the *Bacteroides cellulosolvens* cellulosome reveals novel and distinctive secondary structural elements. J Mol Biol 348:1–12. doi:10.1016/j.jmb.2005.02.024. 15808849

[21] Kearse M, Moir R, Wilson A, Stones-Havas S, Cheung M, Sturrock S, Buxton S, Cooper A, Markowitz S, Duran C, Thierer T, Ashton B, Meintjes P, Drummond A 2012 Geneious basic: an integrated and extendable desktop software platform for the organization and analysis of sequence data. Bioinformatics 28:1647–1649. doi:10.1093/bioinformatics/bts199. 22543367 PMC3371832

[22] Utturkar SM, Klingeman DM, Land ML, Schadt CW, Doktycz MJ, Pelletier DA, Brown SD 2014 Evaluation and validation of *de novo* and hybrid assembly techniques to derive high-quality genome sequences. Bioinformatics 30:2709–2716. doi:10.1093/bioinformatics/btu391. 24930142 PMC4173024

[23] De Leon KB, Utturkar SM, Camilleri LB, Elias DA, Arkin AP, Fields MW, Brown SD, Wall JD 2015 Complete genome sequence of *Pelosinus fermentans* JBW45, a member of a remarkably competitive group of *Negativicutes* in the *Firmicutes* phylum. Genome Announc 2(5):01090-15. doi:10.1128/genomeA.01090-15. PMC4582584 26404608

[24] Woo HL, Utturkar S, Klingeman D, Simmons BA, DeAngelis KM, Brown SD, Hazen TC 2014 Draft genome sequence of the lignin-degrading *Burkholderia* sp. strain lig30, isolated from wet tropical forest soil. Genome Announc 2(3):00637-14. doi:10.1128/genomeA.00637-14. PMC4064042 24948777

[25] Hyatt D, Chen G-L, LoCascio PF, Land ML, Larimer FW, Hauser LJ 2010 Prodigal: prokaryotic gene recognition and translation initiation site identification. BMC Bioinformatics 11:119. doi:10.1186/1471-2105-11-119. 20211023 PMC2848648

[26] Haimovitz R, Barak Y, Morag E, Voronov-Goldman M, Shoham Y, Lamed R, Bayer EA 2008 Cohesin-dockerin microarray: diverse specificities between two complementary families of interacting protein modules. Proteomics 8:968–979. doi:10.1002/pmic.200700486. 18219699

[27] Cameron K, Weinstein JY, Zhivin O, Bule P, Fleishman SJ, Alves VD, Gilbert HJ, Ferreira LMA, Fontes CMGA, Bayer EA, Najmudin S 2015 Combined crystal structure of a type-I cohesin: mutation and affinity-binding studies reveal structural determinants of cohesin-dockerin specificity. J Biol Chem 290:16215–16225. doi:10.1074/jbc.M115.653303. 25934389 PMC4481221

